# A case of closedlip schizencephaly with absent septum pellucidum in an adult presenting with seizure disorder

**DOI:** 10.1002/ccr3.7536

**Published:** 2023-06-13

**Authors:** Chhabi Khadka, Umang Gupta, Prakriti Bhandari, Prabin Pandey, Shailes Paudel

**Affiliations:** ^1^ National Academy of Medical Sciences Kathmandu Nepal; ^2^ Maharajgunj Medical Campus Kathmandu Nepal; ^3^ Patan Academy of Health Sciences Lalitpur Nepal

**Keywords:** closed lip, schizencephaly, seizure, septum pellucidum

## Abstract

**Key Clinical Message:**

To rule out underlying developmental brain defects such as schizencephaly, pediatric seizures necessitate a thorough examination. Adults who receive a diagnosis later in life may face severe management and prognosis difficulties. To avoid underdiagnosis of developing brain abnormalities, imaging should be a part of the workup for pediatric seizures. Imaging is critical to the diagnosis and therapy of such cases.

**Abstract:**

Closed‐lip schizencephaly with the absence of the septum pellucidum is a rare congenital malformation of the brain that can be associated with a variety of neurological conditions. We report the case of a 25‐year‐old male with left hemiparesis who presented with recurrent seizures from childhood, poorly controlled with medications, and increased tremors. He has been taking anticonvulsant for the last 7 years and is under symptomatic management. Magnetic resonance imaging of the brain revealed closed‐lip schizencephaly with absent septum pellucidum.

## Graphical abstract

Normal MRI at the level of optic nerve.

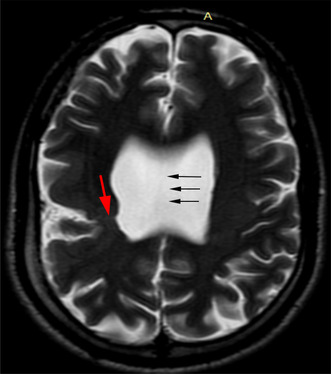

## 1 INTRODUCTION

Schizencephaly is a rare congenital brain malformation characterized by abnormal clefts in the cerebral cortex. It is thought to affect 0.54–1.54 out of every 100,000 live births.^1^ Patients may present with seizures, developmental delay, and motor deficits. Absent septum pellucidum is a common association seen in patients with schizencephaly and has been reported to be present in up to 50% of cases.^2^ Diagnosis is typically made through neuroimaging studies such as magnetic resonance imaging (MRI). We report a 25‐year‐old adult who has an undiagnosed schizencephaly with absent septum pellucidum.

## 2 CASE REPORT

A 25‐year‐old male presented to the outpatient clinic with a chief complaint of increased tremors in both upper extremities, more on the right side, without any weakness. He had a history of recurrent seizures since childhood for which he was under antiepileptic medication (sodium valproate) but was poorly controlled. Appropriate workup on the cause of seizure was not done in the past. There was no significant family history of neurological or psychiatric disorders. There was no history of any birth‐related trauma or maternal exposure to any medications. The patient was unemployed, a non‐smoker, a non‐drinker, and denied any history of recreational drug use.

The patient was alert and oriented on physical examination, with no signs of distress. His vital signs were within normal limits. Neurological examination revealed high‐amplitude moderate frequency resting tremor of both upper extremities with left‐sided weakness (power 4/5 in both left upper and lower limb). Evaluation of tremor was done with recorded video analysis and during office examination. Babinski reflex was negative bilaterally. Considering the possibility of tremors due to valproate, the antiepileptic regimen was switched to levetiracetam.

**FIGURE 1:**
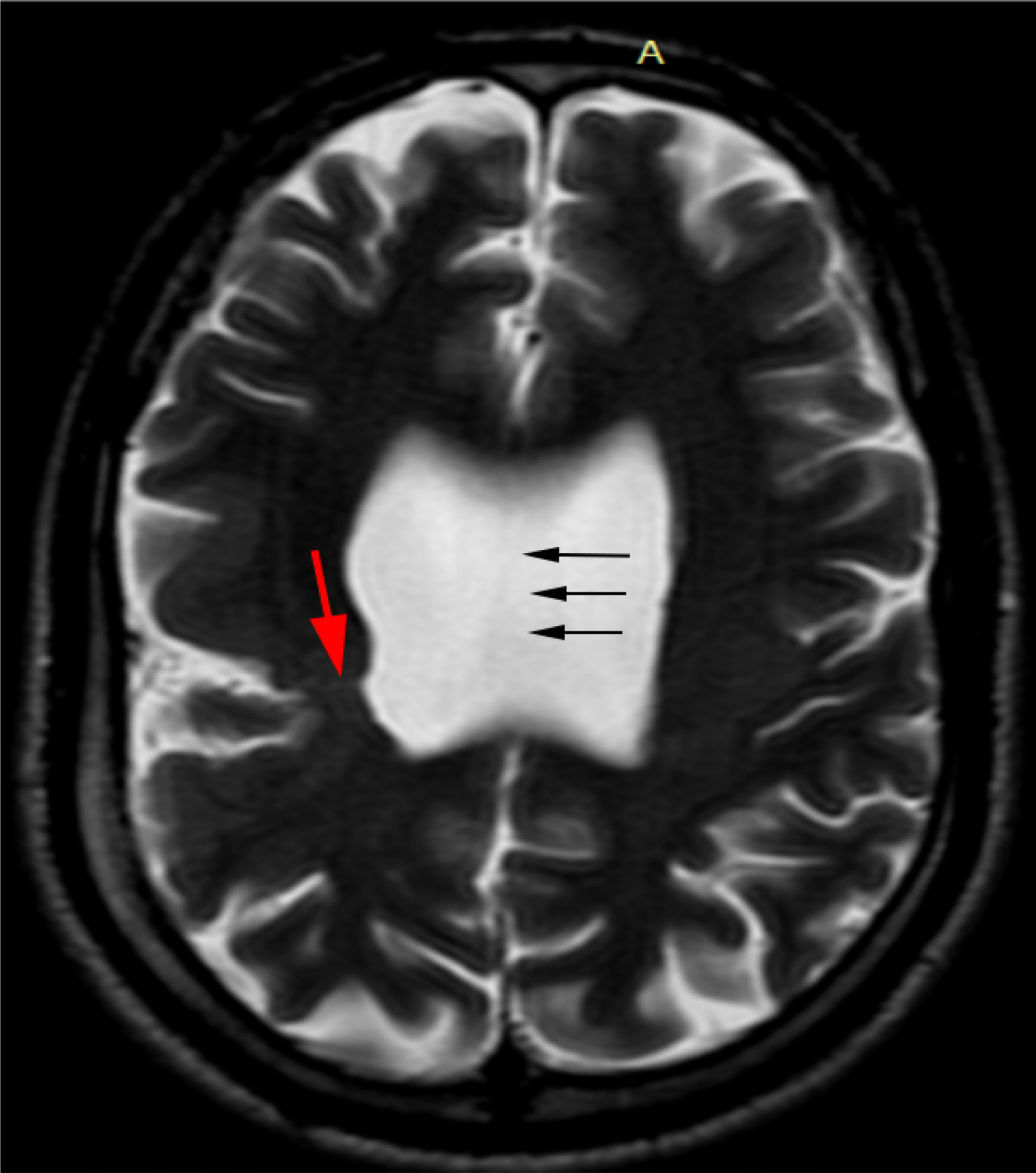
(At the level of lateral ventricle) T2W magnetic resonance imaging axial image shows a subtle T2 hyperintense curvilinear line (red arrow) communicating the lateral ventricle and subarachnoid space of right frontoparietal convexity. Black arrows denote the absence of the septum pellucidum resulting direct communication between the lateral ventricles and squaring off of the frontal horns.

**FIGURE 2:**
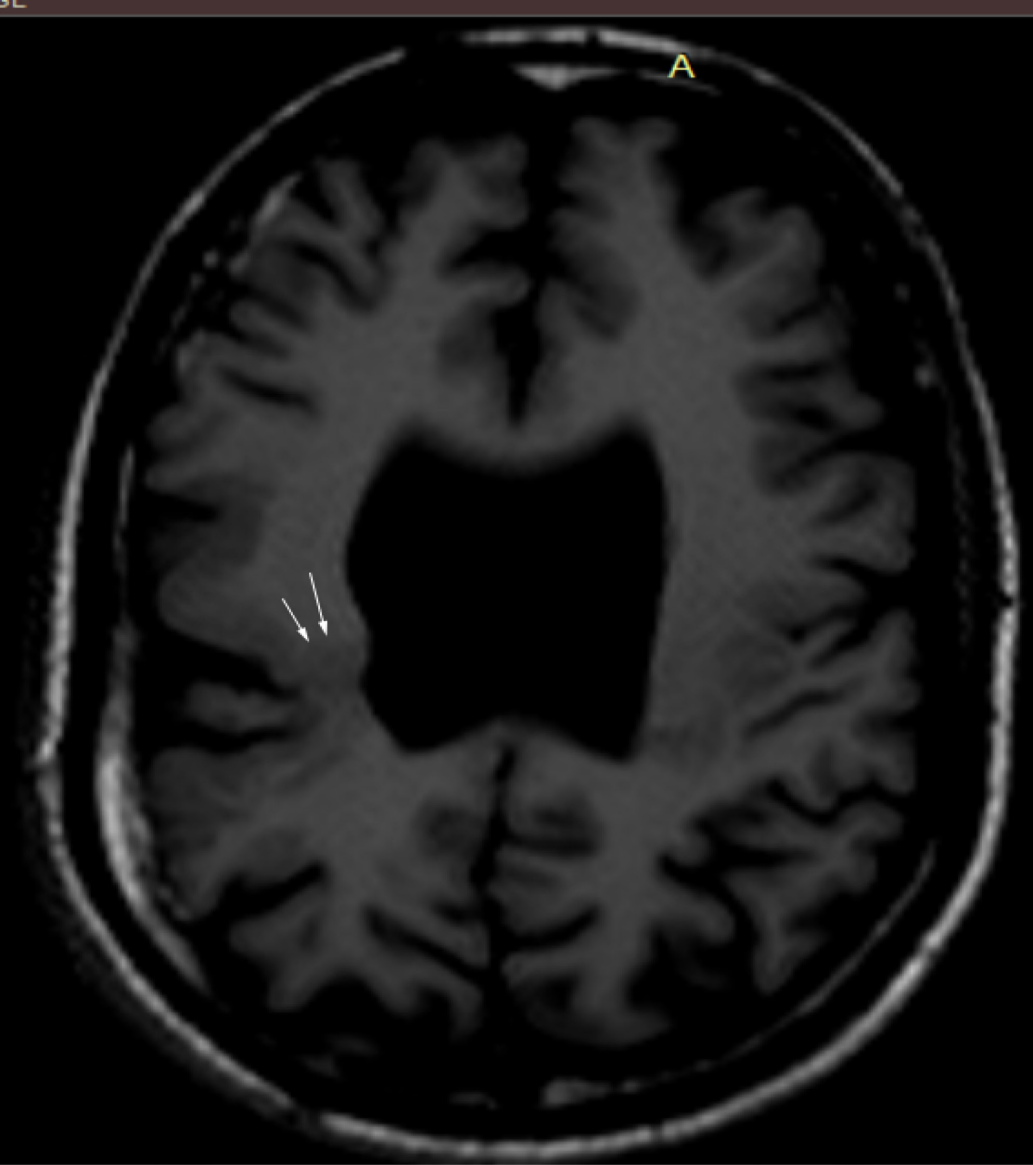
(At the level of lateral ventricle) T1W axial magnetic resonance imaging shows the abnormally located gray matter (white arrows) lining the T2 hyperintense line.

**FIGURE 3:**
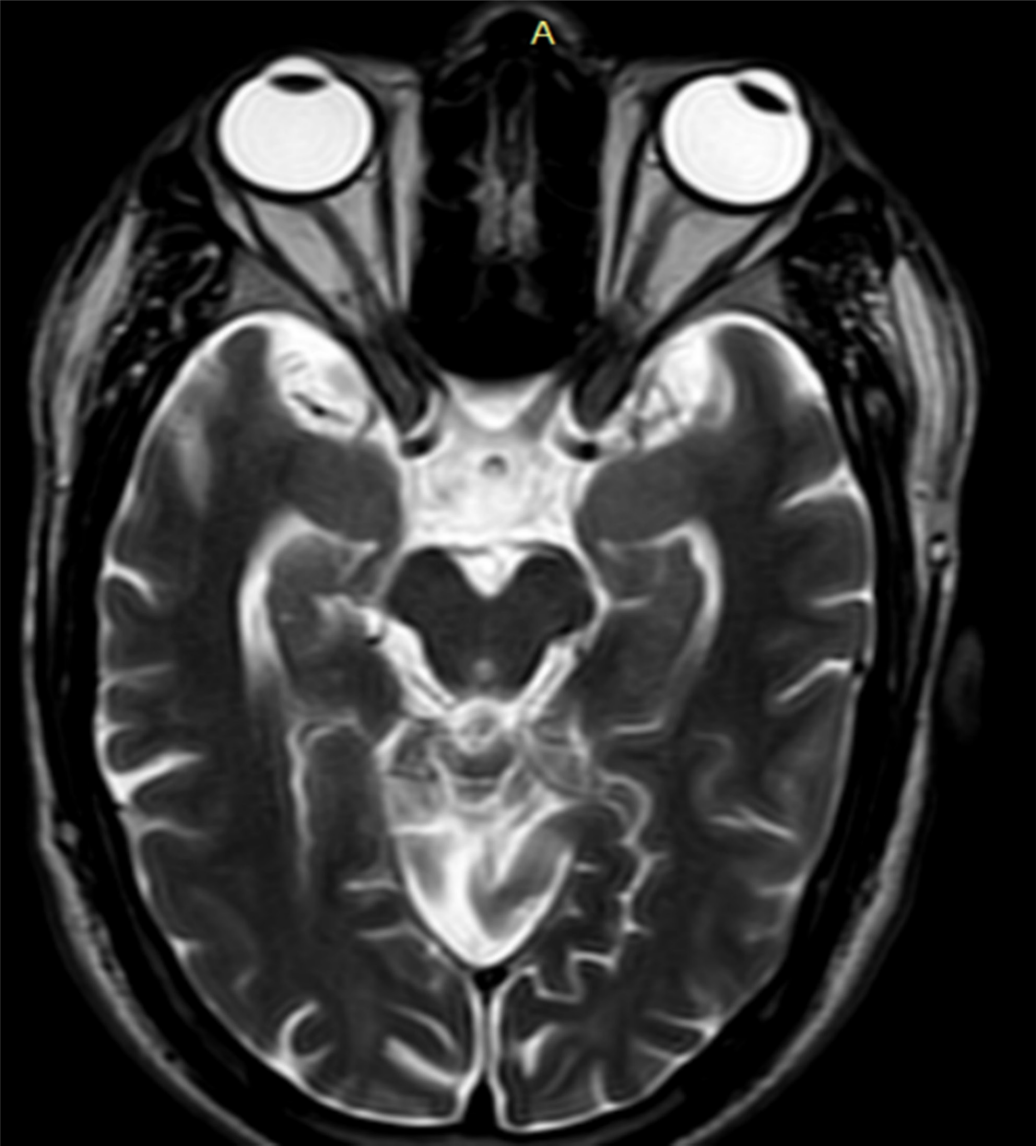
T2W magnetic resonance axial images through the level of optic nerve show their normal size/morphology and normal intensity/morphology of bilateral globes.

MRI brain showed subtle T2 hyperintense curvilinear cleft communicating the lateral ventricle and subarachnoid space of right frontoparietal convexity which is lined by closely opposed gray matter. Absence of the septum pellucidum resulting in direct communication between the lateral ventricles and squaring off of the frontal horns. (Figures 1 and 2). MR axial images through the level of optic nerve show their normal morphology and normal morphology of bilateral globes (Figure 3).

The patient was started on anti‐seizure medication for seizure disorder and referred to a neurologist for further evaluation and management of his tremor. Physical therapy was also initiated to address his left hemiparesis and improve motor function.

Examination on subsequent follow‐up visits showed improvement in tremor and hence the decision to continue levetiracetam was made. The patient has ongoing physical therapy sessions with only slight improvement in his motor function. The patient did not experience any seizure episodes after the medication was changed.

## 3 DISCUSSION

Schizencephaly is a rare congenital neuronal migration disorder characterized by a cleft lined by heterotopic gray matter, which connects the surface of the cerebral hemisphere to the lateral ventricle.^3^ The term was coined by Yakovlev and Wadsworth in 1946, based on their work on cadavers that classified schizencephaly into two types.^4^ These are:

1. Type I (closed‐lip): Cleft is fused, preventing cerebrospinal fluid (CSF) passage.
2. Type II (open‐lip): A cleft is present, which permits CSF to pass between the ventricular cavity and subarachnoid space.

Griffith PD has proposed a new method of classification of schizencephaly based on the appearance of schizencephaly in children and fetuses which led to reassessment of possible etiological and mechanistic causes of schizencephaly.^5^ These are:

1. Type 1 (trans‐mantle): No CSF‐containing cleft on MRI, but contains a trans‐mantle column of abnormal gray matter.
2. Type 2 (closed‐lip): Presence of cleft containing CSF, but the lining lips of abnormal gray matter are abutting and opposed to each other.
3. Type 3 (open‐lip): Presence of cleft containing CSF. The lining lips of abnormal gray matter are not abutting each other.

Developmental delays and a variety of neurological impairments can be linked to both kinds of schizencephaly. The etiopathogenesis of schizencephaly remains unclear, but it may result from external factors like middle cerebral artery stroke or genetic factors like *EMX2* gene mutation. Maternal age, substance abuse, and lack of prenatal care are also potential risk factors.^6^ A related condition is absent cavum septum pellucidum (CSP), which is characterized by the absence or underdevelopment of the cavity between the two lateral ventricles in the brain. In instances of closed lip schizencephaly, the absence of CSP is a frequent finding, and it is frequently employed as a diagnostic standard for the syndrome. With the help of several imaging methods, such as MRI, the absence of the CSP may be shown.^7^ The cleft in the cerebral hemisphere can be seen through imaging tests like an MRI, which are commonly used to diagnose schizencephaly. Managing the neurological abnormalities and developmental delays associated with the illness is often treated with physical, occupational, and speech therapy.^8^

Also, recent research has looked at the use of fiber tractography and diffusion tensor imaging (DTI) in the diagnosis and treatment of brain abnormalities such as schizencephaly.^9^ Although fiber tractography may be used to see the route of important white matter pathways in the brain, DTI is a type of MRI that can give information on the microstructure of brain tissue and the integrity of white matter tracts. These methods could offer insightful details regarding the underlying neurological abnormalities and potential remedies for schizencephalic patients.

DTI and fiber tractography may provide valuable information for the diagnosis and management of these conditions, while genetic testing and molecular profiling may help to identify underlying genetic causes and potential treatment targets.

Some genetic mutations have been reported as possible etiological factors for schizencephaly. The main genes identified in this regard are *COL4A1* mutations, *EMX2*‐germline mutations, *SHH* gene, and *SIX3* gene.^3^ Discrete genetic causes of schizencephaly have been difficult to confirm; hence, no strong evidence exists for an individual's genetic testing.

The usage of valproic acid (VPA), which has been demonstrated to account for 14% of tremors in patients using VPA in a study conducted by Zhang et al., can be linked to the tremor reported in our case.^10^ It has been suggested that the presence of tremors with VPA could be explained by the significant changes in the rate of gamma‐aminobutyric acid (GABA) synthesis in the substantia nigra and corpus striatum. Additionally, disruptions of the GABAergic pathways in the basal ganglia system may cause dopamine inhibition and subsequent changes in catecholamine concentrations.^10^

As the precise source of the disorder is unclear, controlling its symptoms and deficiencies serve as the mainstay of treatment.

## 4 CONCLUSION

To sum up, rare congenital brain anomalies such as closed‐lip schizencephaly may appear in a range of neurological deficits and developmental delays. Like in our instance, such deficits may come to light during maturity as a variety of neurological symptoms. The cause of recurrent neurological symptoms should be identified to determine the best treatment option and prognosis. This should be done whenever feasible in an environment with plenty of resources.

## AUTHOR CONTRIBUTIONS

**Chhabi Khadka:** Conceptualization; formal analysis; project administration; supervision; visualization; writing – original draft; writing – review and editing. **Umang Gupta:** Conceptualization; data curation; methodology; software; validation; visualization; writing – review and editing. **Prakriti Bhandari:** Conceptualization; formal analysis; investigation; project administration; writing – original draft. **Prabin Pandey:** Conceptualization; methodology; supervision; visualization. **Shailes Paudel:** Conceptualization; formal analysis; investigation; methodology; validation; writing – review and editing.

## FUNDING INFORMATION

None.

## CONFLICT OF INTEREST STATEMENT

The authors have no conflict of interest to declare.

## ETHICS STATEMENT

Ethical approval was not required for the case report as per the country's guidelines.

## CONSENT

Written informed consent was obtained from the patient to publish the report.

## Data Availability

The data that support the findings of this study are available from the corresponding author upon reasonable request.
